# Fluorescence-Guided Resection of GL261 Red-FLuc and TRP-mCherry-FLuc Mouse Glioblastoma Tumors

**DOI:** 10.3390/cancers17050734

**Published:** 2025-02-21

**Authors:** Louis T. Rodgers, Bryan J. Maloney, Anika M. S. Hartz, Björn Bauer

**Affiliations:** 1 Department of Pharmaceutical Sciences, College of Pharmacy, University of Kentucky, Lexington, KY 40536, USA; 2 Sanders-Brown Center on Aging, University of Kentucky, Lexington, KY 40536, USA; 3 Department of Pharmacology and Nutritional Sciences, College of Medicine, University of Kentucky, Lexington, KY 40536, USA

**Keywords:** glioblastoma, fluorescence-guided surgery, tumor resection, 5-aminolevulinic acid, GL261 Red-FLuc, TRP-mCherry-FLuc

## Abstract

*Background*: Most preclinical studies on glioblastoma (GBM) fail to provide translational utility in the clinic. Fluorescence-guided surgery using 5-aminolevulinic acid (5-ALA) improves tumor resection, disease prognosis, and, thus, patient outcomes. Given the critical role of surgery in managing recurrent GBM, it is essential to incorporate surgical elements into preclinical models to accurately reflect clinical scenarios and enhance translational success. However, existing protocols for 5-ALA-guided resection in preclinical models are limited and often lack clinical relevance. *Methods*: To address this gap, we developed a novel protocol for the 5-ALA-guided resection in two mouse GBM models: TRP-mCherry-FLuc and GL261 Red-FLuc. *Results*: The resection of TRP-mCherry-FLuc tumors significantly extended survival and mitigated weight loss compared to controls. Similarly, GL261 Red-FLuc tumor resection increased survival, reduced body weight loss, and slowed tumor progression. *Conclusions*: This study presents a clinically relevant protocol for 5-ALA-guided resection in preclinical GBM models, providing a platform for future research to integrate adjuvant therapies and enhance their potential translation into clinical practice.

## Simple Summary

Glioblastoma is a highly aggressive brain tumor with limited treatment options, and most research aimed at improving outcomes has struggled with the translation from laboratory studies to patient care. One major challenge is that existing preclinical models often do not include surgical procedures, even though surgery is a key part of treatment for patients. In this study, we developed and tested a method for the fluorescence-guided resection of glioblastoma tumors in mouse models. Unlike previous models, our approach mimics clinical conditions by targeting tumors located deeper in the brain and by using the FDA-approved imaging agent 5-aminolevulinic acid. By improving the way surgery is incorporated into preclinical models, our method provides a valuable tool for testing new therapies and better understanding tumor behavior after surgery. This work could help bridge the gap between experimental research and clinical outcomes for glioblastoma patients.

## 1. Introduction

Glioblastoma (GBM) is the most common and aggressive primary malignant brain tumor in adults. It is characterized by rapid progression, invasiveness, and resistance to conventional therapies [1]. The current standard of care, established by the 2005 EORTC/NCIC trial, combines maximal safe surgical resection with concurrent radiation therapy and chemotherapy [2]. Despite intensive treatment, GBM patients face a poor prognosis, with a median survival of only 8 months post-diagnosis, irrespective of treatment [1]. Over the past two decades, over 400 clinical trials have been conducted based on promising preclinical data [3]. However, only two phase III trials—CeTeG/NO-09 (lomustine-temozolomide) [4] and EF-14 (tumor treating fields) [5]—reported significant survival benefits [6], highlighting a gap between preclinical efficacy and clinical outcomes. A systematic review of phase I trials from 2006 to 2019 further illustrates this issue, showing that the efficacy observed in preclinical models often fails to translate into meaningful clinical responses, likely due to the challenge of replicating human GBM complexity in animal models [7,8,9,10].

Conventional in vivo preclinical studies commonly use animal models harboring treatment-naïve tumors, thereby failing to replicate the clinical scenario where patients undergo tumor resection followed by radiation and chemotherapy to target residual disease [8]. The therapeutic benefits of maximal safe resection on overall survival in GBM patients have been extensively documented [11,12,13,14,15]. Importantly, surgical intervention directly affects the recurrent disease by triggering processes such as increased cell migration, proliferation, angiogenesis, microglia infiltration, and the upregulation of stem cell markers within recurrent tumors [16,17,18]. Thus, replicating surgical procedures in preclinical models allows for the assessment of the efficacy of adjuvant therapies while accounting for subsequent alterations in the tumor microenvironment, including modifications in vascularization, immune response, and extracellular matrix remodeling. Therefore, integrating surgical interventions into preclinical models more effectively mirrors clinical reality compared to treatment-naïve models, thereby enhancing the potential for translating novel therapeutic approaches into clinical practice.

**Figure 1:**
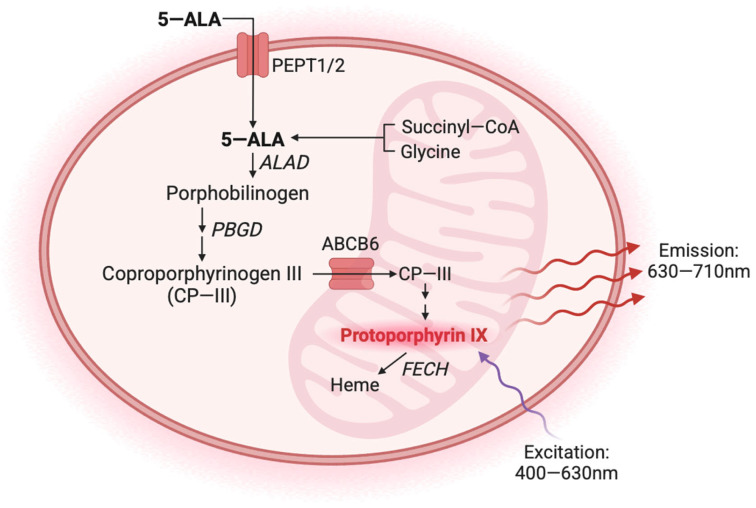
Simplified 5-ALA metabolic pathway. Exogenous 5-ALA enters cells via the transporters peptide transporter 1 (PEPT1) and peptide transporter 2 (PEPT2). Endogenous 5-ALA is synthesized from succinyl-CoA and glycine in the mitochondria. Once inside the cell, 5-ALA is converted into porphobilinogen by ALA dehydratase (ALAD). Porphobilinogen is converted into coproporphyrinogen III through a series of enzymatic reactions beginning with porphobilinogen deaminase (PBGD). Coproporphyrinogen III is translocated from the cytoplasm into the mitochondria by the transporter ABCB6. Protoporphyrin IX (PPIX) is produced following a series of enzymatic reactions and converted into heme by ferrochelatase (FECH). The excitation and emission wavelengths of PPIX, approximately 400–630 nm and 630–710 nm, respectively, contribute to its utility as an optical imaging agent. Figure created with BioRender.com; accessed on 19 February 2025).

In clinical practice, using 5-aminolevulinic acid (5-ALA) during brain tumor surgery has emerged as a promising approach to enhance the extent of tumor resection and subsequently improve patient outcomes [19]. After oral administration, 5-ALA is metabolized within GBM cells via the heme biosynthesis pathway, generating fluorescent protoporphyrin IX (PpIX). PpIX preferentially accumulates in cancer tissue due to variations in enzyme and transporter expression levels and regions of a leaky blood–brain barrier [20,21,22,23] (Figure 1). Upon illumination with 400 nm light, PpIX emits fluorescence, which allows for a real-time intraoperative visualization and delineation of tumor tissue margins during surgery [19]. Thus, fluorescence-guided surgery (FGS) enables neurosurgeons to achieve maximal safe resection while sparing healthy brain tissue.

Current protocols for brain tumor resection in preclinical models, particularly those involving fluorescence-guided techniques, are scarce. Existing fluorescence-guided resection protocols rely on the use of cells labeled with fluorescent tags, such as mCherry, GFP, or RFP, which lack clinical translatability because human tumors are not inherently fluorescent [17,24,25,26,27]. Furthermore, many of these techniques involve the implantation of tumors at shallow depths (0.5–1 mm), failing to replicate the invasive characteristics observed in human tumors. In studies demonstrating 5-ALA fluorescence in preclinical GBM models, 5-ALA imaging was used only for the secondary validation of resection extent, as the tumors were resected using GFP-expressing tumor cells, or survival outcomes were not assessed [25,28,29].

In the present study, we describe a protocol for the 5-ALA-guided resection of two mouse GBM models: TRP-mCherry-FLuc (TRP-mCF) and GL261 Red-FLuc. While GL261 is a widely used syngeneic mouse model, the TRP model originates from a genetically engineered mouse model with mutations in retinoblastoma protein, KRAS, and PTEN [30]. These mutations activate the PI3K pathway, producing tumors that closely resemble patient GBM, exhibiting pseudopalisading necrosis, vessel co-option, and invasion [31]. The resection of TRP-mCF tumors resulted in a significant extension of survival and a slower rate of body weight loss compared to control (no procedure) and sham-resected animals. We did not observe significant differences in survival and weight loss between sham and control mice. Similarly to TRP-mCF tumors, the resection of GL261 Red-FLuc tumors led to increased survival, reduced weight loss, and slower tumor growth compared to control mice. The extent of tumor resection did not significantly impact survival, as over 95% of the tumor was resected in most mice.

## 2. Materials and Methods

### 2.1. Cell Culture

Cell culture was conducted as previously described by Rodgers et al. [31]. Briefly, GL261 Red-FLuc cells were purchased from PerkinElmer (Waltham, MA, USA) and maintained in low-glucose Dulbecco’s Modified Eagle’s Medium (DMEM) supplemented with 10% fetal bovine serum (FBS; VWR, Radnor, PA, USA), and 2 µg/mL puromycin (BioVision, Waltham, MA, USA). TRP-mCF cells were generously provided by Dr. Shawn Hingtgen (University of North Carolina, Chapel Hill, NC, USA) and were maintained in high-glucose DMEM supplemented with 10% fetal bovine serum (FBS; VWR, Radnor, PA, USA) and 1× penicillin–streptomycin (MP Biomedicals, Solon, OH, USA). Both cell lines were cultured at 37 °C and 5% CO_2_. Once at 80–90% confluence, cells were passaged at 80–90% confluence using 0.05% trypsin–EDTA (Corning, Corning, NY, USA), counted using a Scepter 2.0 cell counter (MilliporeSigma, Burlington, MA, USA), and regularly tested for mycoplasma using either the PCR Mycoplasma Test Kit I/C (PromoCell GmbH, Heidelberg, DE) or the MycoStrip™ Mycoplasma Detection Kit (InvivoGen, San Diego, CA, USA).

### 2.2. In Vitro Fluorescence Assay

For in vitro fluorescence assays with 5-aminolevulinic acid (5-ALA, MilliporeSigma, Burlington, MA, USA), 12,500 and 25,000 cells/well were plated in black clear-bottom 96-well plates (Corning, Corning, NY, USA) and incubated overnight at 37 °C and 5% CO_2_. The following day, the cell culture medium was removed, and 200 µL of 5-ALA (1 mM) dissolved in phenol red-free DMEM (Gibco, Thermo Fisher Scientific, Waltham, PA, USA) was added to each well. Fluorescence was measured every 15 min using a Synergy H1 microplate reader (BioTek, Winooski, VT, USA) for 4 h (excitation: 405 nm; emission: 635 nm). Blank wells containing only 1 mM 5-ALA were averaged for each time point and subtracted from wells containing cells. Data were plotted and analyzed using GraphPad Prism^®^ (v9).

### 2.3. Animals

All animal experiments were approved by the University of Kentucky Institutional Animal Care and Use Committee (IACUC #2018-2947; PI: Bauer) and conducted in accordance with the US Department of Agriculture Animal Welfare Act and by the Guide for the Care and Use of Laboratory Animals of the National Institutes of Health.

Seven-week-old female albino B6 (B6(Cg)-*Tyr^c−2J^*/J (Strain No: 000058) (total n = 15) and eight-week-old female homozygous J:NU (Strain No: 007850) (total n = 53) mice were purchased from The Jackson Laboratory (Bar Harbor, ME, USA). Female mice were used to maintain consistency with the gender of the initial tumor host [32,33,34,35]. Mice were group-housed in an AAALAC-accredited temperature- and humidity-controlled facility with controlled temperature (21–22 °C), humidity (30–70%), and a 14:10 h light–dark cycle, and they were provided water and standard chow ad libitum (Envigo Teklad Chow 2918, Envigo, Indianapolis, IN, USA).

### 2.4. Orthotopic Mouse Glioblastoma Models

GBM cell implantations were based on previously published protocols from Carlson et al. for GL261 Red-FLuc [31,36,37] and El Meskini et al. for TRP-mCF [31,38]. Briefly, albino B6 (TRP-mCF model) or J:NU (GL261 Red-FLuc model) mice were prepped with buprenorphine ER-LAB (1 mg/kg, s.c., ZooPharm, Laramie, WY, USA) for analgesia and anesthetized with 2.5% isoflurane using a SomnoSuite^®^ anesthesia system (Kent Scientific, Torrington, CT, USA). Once anesthetized, mice were positioned into a stereotaxic head frame and anesthesia mask (David Kopf Instruments, Tujunga, CA, USA) with a warming pad (Kent Scientific, Torrington, CT, USA), and anesthesia (1–2% isoflurane) was maintained via nose cone. The surgical area was sterilized with alternating swabs of 2% chlorhexidine (Covetrus, Portland, ME, USA) and sterile saline (Covetrus, Portland, ME, USA), and a 1 cm midline incision was made. The periosteum was removed to visualize bregma with 3% H_2_O_2_ (Ward’s Science, Rochester, NY, USA). A 0.9 mm burr hole was drilled at the following coordinates: 2 mm mediolateral and −2 mm anteroposterior from bregma.

For the TRP-mCF model, a 5 µL Hamilton syringe (Hamilton Company, Reno, NV, USA) loaded with a 2500 cells/μL suspension was inserted incrementally at 1 mm/min to a depth of 4 mm, withdrawn 1 mm to create a pocket, and 2 µL was injected over six minutes (0.33 µL/min). The needle was held in place for one minute before being removed incrementally at 1 mm/min. Bone wax (Covetrus, Portland, ME, USA) was shaped into a cone (~1 mm) and placed into the burr hole to prevent any extracranial growth. For the GL261 Red-FLuc model, a 5 µL Hamilton syringe loaded with a 2500 cells/μL suspension was inserted to a depth of 4 mm, withdrawn 1 mm, and 2 µL was injected over two minutes (1 µL/min). The needle remained for one minute before it was removed.

Leakage or blood at the injection site was cleaned with ethanol-soaked cotton applicators, and the burr hole was sealed with melted bone wax. The incision was closed with 9 mm wound clips (Fine Science Tools, Foster City, CA, USA), and mice were transferred to a recovery cage on a heating pad (Stryker, Kalamazoo, MI, USA). Mice were monitored for at least 3 h post-surgery until they exhibited normal behavior, including regular grooming, exploratory activity, and ambulation without signs of distress. Mice were monitored daily for signs of distress or endpoint criteria, including 25% body weight loss, behavior changes, imbalance, head tilt, or altered respiration [39,40].

### 2.5. Bioluminescence Imaging

In vivo bioluminescence imaging was conducted as previously described [31]. Imaging was conducted weekly on GL261 Red-FLuc mice to monitor tumor take and growth. Mice received XenoLight^®^ RediJect™ D-luciferin (150 mk/kg, 5 µL/g; i.p., PerkinElmer, Waltham, MA, USA) and were anesthetized with 2% isoflurane. After 10 min, tumor bioluminescence was measured using a Lago in vivo optical imaging system (Spectral Instruments Imaging, Tucson, AZ, USA; FOV: 21.6 cm, f-stop: 2, binning: 4) and analyzed with Aura Imaging 4.0.7 software (Spectral Instruments Imaging, Tucson, AZ, USA).

### 2.6. T2-Weighted Magnetic Resonance Imaging

Two weeks post-implantation, we confirmed TRP-mCF tumor engraftment using T2-weighted magnetic resonance imaging (MRI). Mice were anesthetized with 1.5–2% isoflurane and imaged using a 7T Bruker BioSpec small animal MRI scanner (Bruker BioSpin, Billerica, MA, USA). Temperature and respiratory rate were monitored, T2-weighted images were obtained (TR: 4000 ms, TE: 33 ms, FOV: 20 × 20 × 8), and then mice recovered in a warmed cage before being returned to their home cage. Images were analyzed using *syngo.via* VB60A_HF07 software (Siemens Medical Solutions USA, Inc., Malvern, PA, USA).

### 2.7. Fluorescence-Guided Tumor Resection

**Figure 2:**
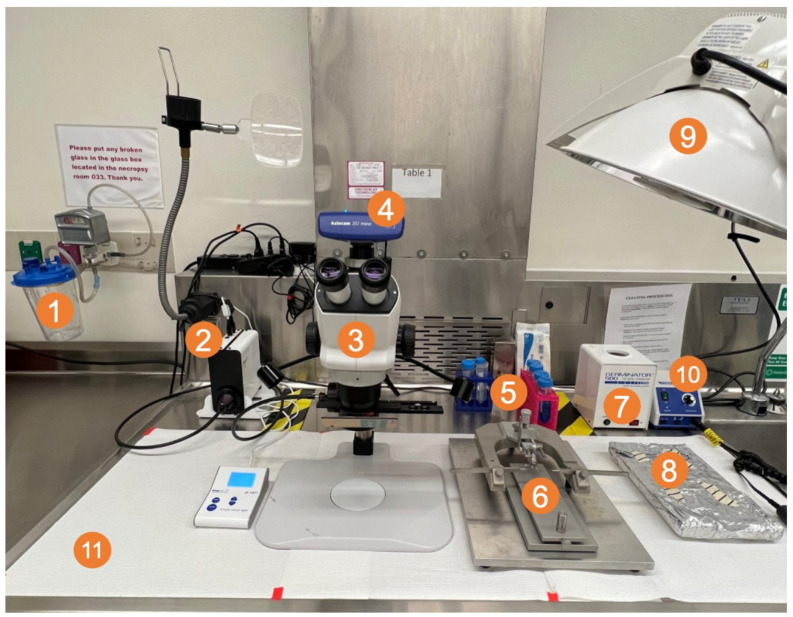
Surgical field setup with (1) vacuum trap; (2) CoolLED pE300 multiband LED light source; (3) ZEISS Stemi 508 stereo microscope with chroma filter set AT425/50x, AT485DC, AT655/30m; (4) ZEISS Axiocam 202 mono microscope camera; (5) sterile Pasteur pipettes, cotton-tipped applicators, and tubes of 2% chlorhexidine, saline, 70% ethanol, and 3% hydrogen peroxide; (6) small animal stereotaxic frame with ear bars and adjustable platform; (7) bead sterilizer; (8) sterile surgical equipment (scalpel, forceps, drill bit, staples, staple applicator) (9) light source; and (10) micromotor kit with rotary handpiece, control box, and foot pedal (pedal under table; not shown in picture).

**Figure 3:**
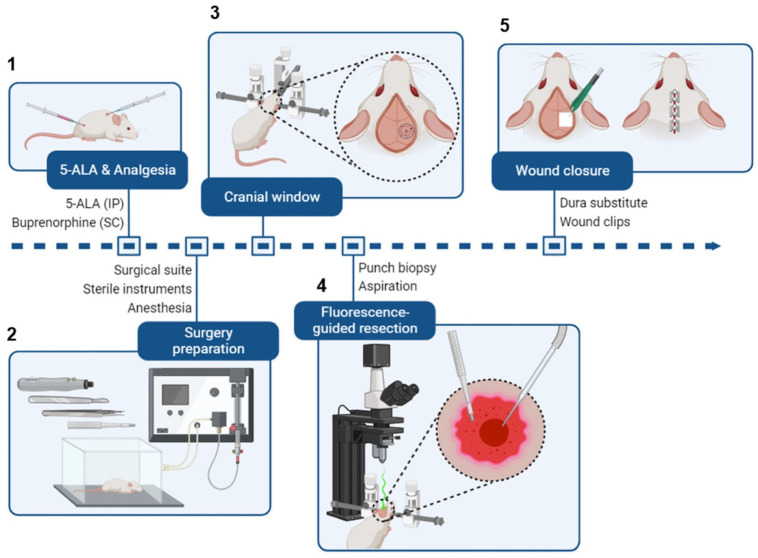
Brief schematic of intracranial tumor resection from a mouse. (**1**) Mice receive 5-ALA (i.p.) and buprenorphine (s.c.) 2 h before surgery. (**2**) Preceding the surgery, the surgical area is organized (see Figure 2). Mice are administered isoflurane in an induction chamber. (**3**) Once the mice are fully anesthetized, they are transferred to a stereotaxic frame, and the surgical site is sterilized. The scalp is cut to expose the previous burr hole, and a cranial window is created around it. (**4**) A 2 mm (diameter) × 3 mm (depth) portion of the tumor is excised using a punch biopsy tool or sample corer. Then, a fluorescence-capable stereo microscope is used to detect any residual fluorescent tumor tissue, which is removed by aspiration. (**5**) The cranial window is closed with a dura substitute to preserve intracranial pressure, and the wound is closed with wound clips. Figure created with BioRender.com; accessed on 2 April 2024.

On the day before resection, tumors were verified using in vivo bioluminescence imaging (GL261 Red-FLuc) or T2-weighted MRI (TRP-mCF). Then, mice with tumors were randomized into groups (e.g., control, sham, or resection), and albino B6 mouse heads were shaved. A picture of the surgical setup and a brief schematic of the procedure are shown in Figure 2 and Figure 3, respectively. The preparatory and positioning procedures for the animals closely mirrored those detailed in the intracranial injection procedure. On the morning of the resection, albino B6 (TRP-mCF model; day 15 post-implantation) or J:NU (GL261 Red-FLuc model; day 14 post-implantation) mice were injected with buprenorphine ER-LAB (1 mg/kg, s.c.). Two hours before the start of the resection, mice were injected with 5-ALA (500 mg/kg; saline; i.p.). In an induction chamber, mice were anesthetized with 2.5% isoflurane using a SomnoSuite^®^ anesthesia system. Once under anesthesia, mice were transferred to a platform with an infrared warming pad and positioned into a stereotaxic head frame and anesthesia mask. Isoflurane was switched from the induction chamber to the nose cone and maintained at 1–2% for the procedure. Alternating swabs of 2% chlorhexidine and sterile saline were used to sterilize the animal’s head. Following sterilization of the surgical area, a 1 cm midline incision was made using a 22-blade sterile disposable scalpel, and the skin was retracted using mini-Colibri retractors (Fine Science Tools, Foster City, CA, USA). The periosteum was gently removed with cotton-tipped applicators, and the previous burr hole was visualized. A circular cranial window of about 2.5 mm in diameter was created by thinning the skull with an MH-170 rotary handpiece and 0.9 mm micro drill burr. Once the skull was thin enough, 1–2 drops of saline were added before gently lifting the inner portion. Blood and excess saline were removed with sterile cotton tips. An autoclaved reusable 2 mm sample corer (Fine Science Tools, Foster City, CA, USA) was inserted into the established cranial window to a depth of 2 mm. The corer was gently twisted for 10 s to separate the tissue and then gently removed. Using a ZEISS Stemi 508 Stereo Microscope with chroma filter set AT425/50x, AT485DC, AT655/30m (Carl Zeiss AG, Oberkochen, Germany), the fluorescent tumor tissue was identified, and the biopsy was gently aspirated with a Pasteur pipette attached to a medical vacuum system (BeaconMedaes, Rock Hill, SC, USA). Remaining fluorescent tissue and blood were removed gently with suction alternating between fluorescence guidance and white light. Gentle pressure was applied over the resection site using a sterile cotton-tipped applicator until any active bleeding subsided. To recreate a closed system, the cranial window was covered with a 3 mm × 3 mm piece of Neuro-Patch^®^ dura substitute (Aesculap, Inc., Center Valley, PA, USA), which was sealed using veterinary surgical adhesive (Covetrus, Portland, ME, USA) and bone wax. The skin was closed with 9 mm wound clips, and the mouse was moved to a clean cage on a heating pad. A Petri dish with moistened food was placed on the cage bottom to aid recovery. Mice were closely monitored for at least 3 h post-surgery until they exhibited normal behavior, including regular grooming, exploratory activity, and ambulation without signs of distress. Following injections, daily observations continued until they reached a humane endpoint, including a 25% body weight loss or signs of altered behavior, imbalance, head tilt, or respiration. Sham mice underwent the same resection procedure (administration of buprenorphine, 5-ALA, and isoflurane; craniectomy; and Neuro-Patch^®^ placement) except for the removal of brain tissue. Control mice received no intervention.

### 2.8. Data Analysis and Statistics

Data were analyzed by generalized mixed-level linear models for all data, except that Cox survival models were applied to survival times, and joint models for combined longitudinal and time-to-event data [41] were applied to mean change in body weight and mean post-resection luminescence vs. time. Joint modeling was conducted for time points beginning on the day of resection and ending on the last day when more than one animal was alive in a group. Nakagawa’s coefficient of determination (*R*^2^) was used [42] for all models except for Cox models, where Nagelkerke’s *R*^2^ [43] was used. Comparing more than two pairwise differences was challenging since no formal method is known for such comparisons in joint models. Therefore, pairwise models were also constructed, and *p* values for hazard ratios and longitudinal trends were adjusted by the Benjamini–Hochberg false discovery rate [44]. Analysis was performed with the R statistical environment (4.2.2) [45] and the JM (1.5-2) [46], lme4 (1.1-35.5) [47], performance (0.12.4) [48], and survival (3.7-0) [49] packages.

## 3. Results

### 3.1. In Vitro 5-ALA-Induced Fluorescence of GL261 Red-FLuc and TRP-mCF Cells

**Figure 4:**
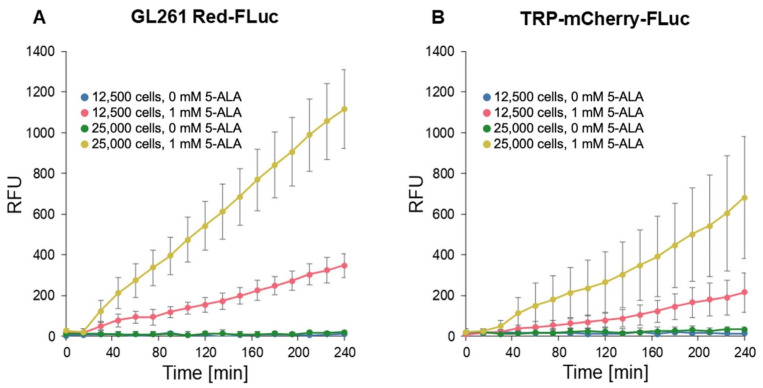
In vitro fluorescence of GL261 Red-FLuc and TRP-FLuc following incubation with 5-ALA. (**A**) Quantified in vitro fluorescence of 12,500 and 25,000 GL261 Red-FLuc cells over four hours after adding either 0 mM or 1 mM 5-ALA (n = 6, 3 technical replicates; data points represent mean ± SEM). (**B**) Quantified in vitro fluorescence of 12,500 and 25,000 TRP-mCF cells over four hours after adding either 0 mM or 1 mM 5-ALA (n = 6, 3 technical replicates; data points represent mean ± SEM). As described in the text, 5-ALA treatment significantly increased the rate of RFU increase over time, as did higher seeding count. Errors are SEM calculated by pooled variances.

We conducted fluorescence imaging to confirm in vitro porphyrin accumulation in GL261 Red-FLuc and TRP-mCF cells (Figure 4). Cells were seeded at densities of 12,500 and 25,000 cells/well and allowed to settle overnight. Subsequently, 1 mM 5-ALA was added, and fluorescence intensity [Relative Fluorescence Unit (RFU); Ex: 405 nm; Em: 635 nm)] was measured every 15 min over 4 h. The fluorescence intensity of wells exposed to 5-ALA exhibited a significant increase over time, as indicated by the slope of RFU/time, compared to untreated cultures (Figure 4, Table 1). This enhancement was observed in both cell types, and fluorescence varied significantly based on cell seeding density. We began analysis at the 15 min time point due to a discernible lag in signal change between 0 and 15 min. Appropriate pairwise comparisons (Table 2) showed that 5-ALA significantly enhanced signal development over time, and the initial cell count also significantly increased fluorescence. Additionally, GL261 Red-FLuc cells exhibited a higher signal compared to TRP-mCF cells.

### 3.2. Resection of TRP-mCF Tumors Significantly Extended Mouse Survival and Slowed Body Weight Loss

**Figure 5:**
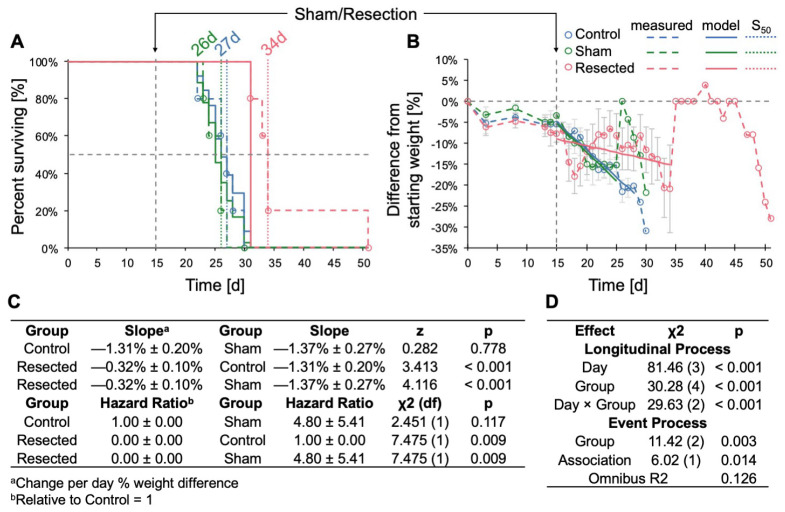
Effects of TRP-mCF tumor resection on survival and body weight change. Animals were implanted with 5000 TRP-mCF cells on day 0, and tumors were resected in one third of the animals on day 15 (n = 5/group). (**A**) Survival curve of control, sham, and resection TRP-mCF mice with median survivals of 27, 26, and 34d, respectively. Analysis of survival using a Cox model demonstrated that resection enhanced median survival. The dotted line corresponds to the day of sham or resection procedures (d15). (**B**) Normalized (% of day 0) weight of control, sham, and resection mice. The dotted line corresponds to the day of sham or resection procedures (d15). Following tumor resection, mice exhibited initial weight loss compared to control and sham-operated groups, with subsequent weight recovery observed by day 3 post-surgery. (**C**) Estimated marginal trends (slopes) for weight change and hazard ratios for survival. (**D**) Analysis of % weight change from day 0 by joint longitudinal and time-to-event model, showing that resection significantly reduced the rate of weight loss.

When evaluating the impact of resection across control, sham-resected, and resected TRP-mCF animals, we observed a significant increase in survival following resection, with median survival durations of 27, 26, and 34d, respectively (*p* < 0.001) (Figure 5A). In addition, the rate of body weight loss, assessed as the percent difference from day 0, was significantly slower in resected animals compared to both control and sham-operated animals (Figure 5B). A transient decrease in mean weight was observed in mice who underwent resection for the two days following resection; however, these mice began to regain weight by the third day after the procedure. In contrast, no post-surgical weight loss was observed in the sham-operated mice. Of note, the abrupt increases in weight observed in both sham and resected mice on days 26 and 35 were attributed to the loss of mice with 25% weight loss nearing the experimental endpoint, with the remaining animals’ weights contributing to sharp rises in the group average.

### 3.3. Resection of GL261 Red-FLuc Tumors Extended Survival, Reduced Body Weight Loss, and Slowed Tumor Growth

**Figure 6:**
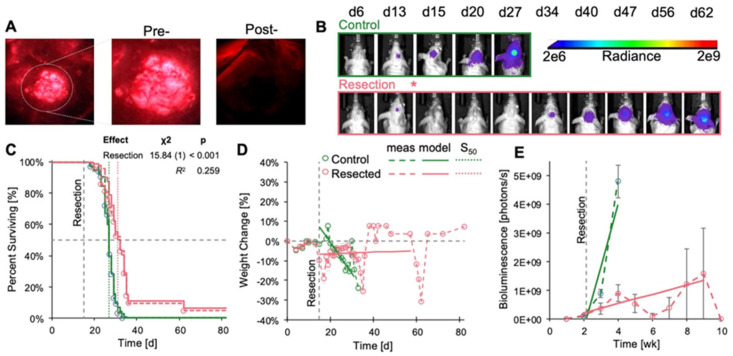

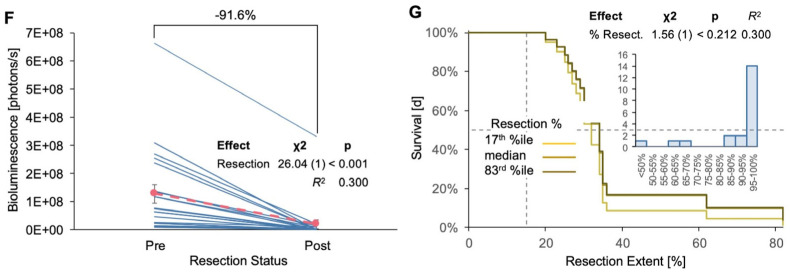
Effects of GL261 Red-FLuc tumor resection on survival, body weight change, and tumor growth. Animals were implanted with 5000 GL261 Red-FLuc cells on day 0, and tumors were resected on day 14 (n = 32: control, 21: resection). (**A**) Representative image of fluorescent tumor tissue pre- and post-resection. (**B**) Representative in vivo bioluminescence images in J:NU mice intracranially injected with 5000 GL261 Red-FLuc cells. Resections were conducted 14 days post-implantation, while control mice received no intervention. Total emission range: 2 × 10^6^ to 2 × 10^9^ photons/s. (**C**) Survival curve of control and resection GL261 Red-FLuc mice with median survivals of 27 and 32d, respectively. Survival analysis using the Cox model demonstrated that resection enhanced median survival. The dotted line corresponds to the day of sham or resection procedures (d14). One resection animal was euthanized after 12 weeks due to the absence of recurrent tumor evidence on bioluminescence imaging. (**D**) Normalized (% of day 0) weight of control and resection mice. The horizontal dotted line corresponds to the day of resection (d14). Analysis of % weight change from day 0 by joint longitudinal and time-to-event model showed that resection significantly reduced the rate of weight loss. (**E**) Average quantified tumor bioluminescence by week, demonstrating a significant difference in bioluminescence between control and resection mice beginning at week three post-implantation (one-week post-resection). (**F**) Quantified tumor bioluminescence on day 13 (one day pre-resection) and day 15 (one day post-resection) for resection mice, demonstrating an average resection extent of 91.6% (*p* < 0.001). Pink corresponds to average values. (**G**) An evaluation of the impact of extent of resection on survival outcomes.

We also resected tumors in GL261 Red-FLuc mice and monitored their survival, body weight loss, and relative tumor growth (Figure 6). Sham-operated animals were omitted from this study as there was no difference in survival or rate of body weight loss compared to controls (Figure 5). Representative images depicting 5-ALA-induced fluorescence of tumor pre- and post-resection, along with the temporal progression of tumor bioluminescence, are illustrated in Figure 6A,B, respectively. Resection resulted in a significant increase in survival compared to unresected controls (27 vs. 32d; *p* < 0.001) (Figure 6C). Additionally, weight loss in resected animals was significantly slower than in unresected animals (Figure 6D, Table 3). Similar to observations in TRP-mCF mice, the abrupt increases in weight towards the end of the study were attributed to the loss of mice with 25% weight loss nearing the experimental endpoint, with the remaining animals’ weights contributing to sharp rises in the group average. Therefore, we caution against overinterpreting the predicted trend for weight change in resected animals, as median survival for resected animals was at 32 days. As time progressed, fewer animals survived, enhancing any survivor effect on the overall trend. While tumor (re)growth was slower in resected animals (Figure 6E, Table 4), the association coefficient was not significant, indicating that the event process (death) did not significantly influence the trajectory of the longitudinal process (luminescence) in this context. Regarding the extent of resection, bioluminescence levels on the day following resection exhibited a significant decrease compared to corresponding pre-resection values, with an average reduction in tumor bioluminescence of 91.6% (Figure 6F). Finally, we explored whether the extent of resection impacted survival (Figure 6G), a factor demonstrated in clinical studies [12,13,14]. However, we observed no significant effect, which was anticipated given that 86% of mice underwent resection of at least 85% of the tumor, and 67% had tumor resection exceeding 95% (Figure 6G inset). Overall, these findings emphasize the survival advantage conferred by tumor resection and reinforce the importance of surgical intervention in preclinical GBM models.

## 4. Discussion

The gap between promising preclinical GBM studies and their limited clinical translation remains a significant challenge in advancing patient outcomes. Despite the insights provided by preclinical models, their applicability and translatability to clinical settings often fall short [10]. As the impact of surgery on recurrent GBM becomes increasingly evident [16,17,18], incorporating surgical interventions into preclinical models, as demonstrated in our study, has the potential to narrow this gap and enhance translational efficacy.

Despite mounting evidence highlighting the significance and impact of surgery in GBM, preclinical models incorporating resection procedures remain limited. Existing protocols for punch biopsy or fluorescence-guided surgery using labeled cells offer certain advantages but also present limitations [25,26,27,50]. Punch biopsy, for instance, provides a faster but less invasive method for excising a section of tumor tissue compared to alternative approaches involving visualization paired with aspiration or microdissection [50]. However, punch biopsy procedures result in variable amounts of residual tumor tissue, leading to inconsistencies in survival outcomes among treatment groups.

On the other hand, fluorescence-guided surgery closely simulates maximal safe surgical resection performed in patients, making it a promising technique. However, most protocols for fluorescence-guided resection in preclinical models rely on labeled cells with limited clinical relevance [25,26,27]. Moreover, these procedures typically implant tumors at shallow depths (0.5–1 mm) and are left as open systems without closure of the craniotomy. These aspects fail to replicate the invasiveness of patient tumors [51] and the normalization of intracranial pressure observed in clinical settings [52,53]. While some groups have explored the use of alternative fluorescent imaging agents, such as sodium fluorescein [54], 5-ALA is currently the only FDA-approved imaging agent for glioma surgery [55]. Previous studies have demonstrated the visualization of GBM tumors in preclinical models using 5-ALA, but these investigations primarily served as proof-of-principle studies and did not assess animal survival [28,29].

Our protocol offers several advantages, including using 5-ALA, validation across two distinct mouse GBM models with differing phenotypes, targeting deep-seated tumors, and adopting a closed system approach, which may effectively mitigate potential confounding variables. Interestingly, our protocol demonstrated a slower regrowth of recurrent tumors compared to untreated controls, contrasting with previous observations of rapid growth following resection in other studies [17,25]. This discrepancy could stem from differences in resection procedures, such as our use of a closed system and the resulting maintenance of intracranial pressure, the implantation of deep-seated tumors, or model-specific factors; however, further investigation would be necessary to elucidate this difference.

On the other hand, it is also important to acknowledge the limitations of our technique, including the absence of other components of the standard of care, which have also been shown to affect recurrent disease [56,57], and the time-intensive and invasive nature of the procedure, which may contribute to initial body weight loss in animals. Furthermore, 5-ALA is considered a nonspecific imaging agent, and fluorescence could exhibit greater variability compared to labeled cells; however, studies have demonstrated an overlap between 5-ALA fluorescence and GFP-labeled tumors [25]. Additionally, 5-ALA fluorescence was dim in deeper brain regions and challenging to distinguish in active bleeding, leading to prolonged operation times. Lastly, this study included only female mice. While it is customary to implant GL261 cells in female mice, tumor establishment has also been observed in male mice, which exhibit a more tumor-supportive microenvironment compared to females [58].

Despite near-maximal tumor resection in most animals, our results demonstrated only a modest survival benefit and no significant correlation between extent of resection and survival, highlighting the challenges of GBM biology and preclinical models. The survival benefit observed in our study—approximately 22% of the median survival of untreated animals—is comparable to the survival advantage seen in the Stupp trial, where temozolomide and radiation therapy (median survival: 14.6 months) increased survival by about 21% compared to radiation alone (median survival: 12.1 months) [2]. These findings suggest that multimodal therapeutic strategies, such as combining fluorescence-guided resection with immunotherapy, targeted treatments, or radiotherapy, may be essential to improve the predictive power of preclinical GBM models. Additionally, using alternative tumor models, particularly those characterized by slower tumor growth, may offer enhanced predictive value and greater clinical relevance. While surgical steps in preclinical models ensures the incorporation of resection-related variables, further improvement of these models and the integration of multimodal strategies may be necessary to address the challenges of clinical translation.

Despite these considerations, our technique presents an innovative approach to fluorescence-guided tumor resection in preclinical models, offering valuable insights into recurrent tumor growth dynamics and informing the potential efficacy of adjuvant therapies.

## 5. Conclusions

Our study underscores the critical need for improved preclinical models in GBM research to bridge the gap between experimental findings and clinical outcomes. Despite the growing recognition of the pivotal role of surgery in GBM management, existing preclinical protocols for tumor resection remain limited in their translational relevance and applicability. Our innovative approach, utilizing 5-ALA-guided resection validated across two distinct mouse GBM models, addresses some limitations by targeting deep-seated tumors and adopting a closed-system approach. Our protocol offers a promising approach to studying GBM and evaluating novel therapeutic strategies.

## Figures and Tables

**Table 1:** In vitro fluorescence of GL261 Red-FLuc and TRP-mCF cells with 5-ALA.

Effect	χ^2^ (df)	*p*	*R* ^2^
5-ALA	326 (1)	<0.001	0.512
Time	1337 (1)	<0.001	0.268
Cells	104 (1)	<0.001	0.241
Line	28 (1)	<0.001	0.085
5-ALA × Time	1285 (1)	<0.001	0.131
5-ALA × Cells	97 (1)	<0.001	0.117
Time × Cells	392 (1)	<0.001	0.060
5-ALA × Line	35 (1)	<0.001	0.046
Cells × Line	9 (1)	0.002	0.020
Time × Line	88 (1)	<0.001	0.018
5-ALA × Time × Cells	361 (1)	<0.001	0.029
5-ALA × Cells × Line	10 (1)	0.002	0.011
5-ALA × Time × Line	93 (1)	<0.001	0.009
Time × Cells × Line	28 (1)	<0.001	0.005
5-ALA × Time × Cells × Line	33 (1)	<0.001	0.002
Overall	2282 (15)	<0.001	0.761

**Table 2:** Relevant pairwise comparisons of the rate of in vitro fluorescence generation.

Cells	5-ALA	RFU/min	Cells	5-ALA	RFU/min	t (df)	*p*
GL261 Red-FLuc							
12,500	0	0.001 ± 0.019	25,000	0	0.005 ± 0.019	0.164 (1448)	0.869
12,500	0	0.001 ± 0.019	12,500	1	0.276 ± 0.019	10.360 (1448)	<0.001
25,000	0	0.005 ± 0.019	25,000	1	0.935 ± 0.019	35.064 (1448)	<0.001
12,500	1	0.276 ± 0.019	25,000	1	0.935 ± 0.019	24.868 (1448)	<0.001
TRP-mCF							
12,500	0	−0.002 ± 0.042	25,000	0	0.015 ± 0.042	35.064 (1448)	0.869
12,500	0	−0.002 ± 0.042	12,500	1	0.169 ± 0.042	24.868 (1448)	0.005
25,000	0	0.015 ± 0.042	25,000	1	0.537 ± 0.042	9.087 (36,192)	<0.001
12,500	1	0.169 ± 0.042	25,000	1	0.537 ± 0.042	2.605 (36,192)	<0.001

**Table 3:** GL261 Red-FLuc tumor resection effects on body weight.

Effect	χ^2^ (df)	*p*
Longitudinal Process		
Day	111.85 (2)	<0.001
Group	134.43 (2)	<0.001
Day × Group	111.43 (1)	<0.001
Event Process		
Group	5.48 (1)	<0.019
Association	35.78 (1)	<0.001
Omnibus *R*^2^		0.105

**Table 4:** GL261 Red-FLuc tumor resection effects on bioluminescence.

Effect	χ^2^ (df)	*p*
Longitudinal Process		
Day	154.98 (2)	<0.001
Group	165.29 (2)	<0.001
Day × Group	111.69 (1)	<0.001
Event Process		
Group	15.04 (1)	<0.001
Association	0.09 (1)	0.769
Omnibus *R*^2^		0.049

## Data Availability

The original contributions presented in this study are included in the article. Further inquiries can be directed to the corresponding author.
